# Molecularly Imprinted Titanium Dioxide: Synthesis Strategies and Applications in Photocatalytic Degradation of Antibiotics from Marine Wastewater: A Review

**DOI:** 10.3390/ma18092161

**Published:** 2025-05-07

**Authors:** Xue Han, Yu Jin, Luyang Zhao, Yuying Zhang, Binqiao Ren, Xiaoxiao Song, Rui Liu

**Affiliations:** 1 Institute of Advanced Technology, Heilongjiang Academy of Sciences, Harbin 150009, China; hxyep883@163.com (X.H.); jinyu36@126.com (Y.J.); zly516678@163.com (L.Z.); 18847008955@163.com (Y.Z.); 2 Heilongjiang Institute of Environmental and Sciences, Harbin 150056, China; 3 Center of Pharmaceutical Engineering and Technology, Harbin University of Commerce, Harbin 150076, China

**Keywords:** molecularly imprinted, TiO_2_, photocatalysis, antibiotics

## Abstract

Antibiotic residues in the marine environment pose a serious threat to ecosystems and human health, and there is an urgent need to develop efficient and selective pollution control technologies. Molecular imprinting technology (MIT) provides a new idea for antibiotic pollution control with its specific recognition and targeted removal ability. However, traditional titanium dioxide (TiO_2_) photocatalysts have limited degradation efficiency and lack of selectivity for low concentrations of antibiotics. This paper reviews the preparation strategy and modification means of molecularly imprinted TiO_2_ (MI-TiO_2_) and its composites and systematically explores its application mechanism and performance advantages in marine antibiotic wastewater treatment. It was shown that MI-TiO_2_ significantly enhanced the selective degradation efficiency of antibiotics such as tetracyclines and sulfonamides through the enrichment of target pollutants by specifically imprinted cavities, combined with the efficient generation of photocatalytic reactive oxygen species (ROS). In addition, emerging technologies such as magnetic/electric field-assisted catalysis and photothermal synergistic effect further optimized the recoverability and stability of the catalysts. This paper provides theoretical support for the practical application of MI-TiO_2_ in complex marine pollution systems and looks forward to its future development in the field of environmental remediation.

## 1. Introduction

In recent years, the development of efficient and selective pollutant degradation technologies has become a research hotspot as the problem of antibiotic pollution in the marine environment has become increasingly serious. Antibiotic wastewater enters the marine environment through industrial discharges, agricultural runoff, and atmospheric deposition, posing a serious threat to marine ecosystems and human health [1]. The World Health Organization (WHO) announced in 2019 that antimicrobial drug resistance is one of the top ten current threats to global health [2]. The overuse of antibiotics has led to the prevalence of antibiotic resistance. Global per capita antibiotic consumption in an estimated 76 countries was reported to have increased by 65% from 2000 to 2015 [3], and the COVID-19 pandemic has further exacerbated the problem of antibiotic misuse. Conventional treatment technologies (e.g., adsorption, biodegradation) have difficulty coping with the complex challenges of high salinity and multi-pollutant coexistence in the marine environment, and, therefore, the development of novel degradation technologies combining high selectivity, resistance to interference, and environmental adaptability is imminent [4].

**Figure 1:**
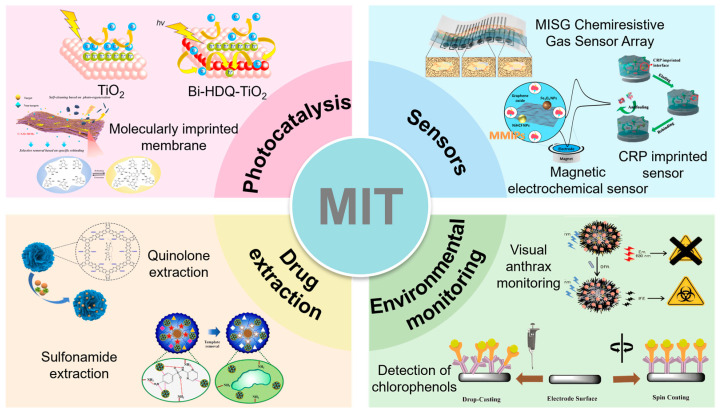
Application fields related to MIT [6,7,8,9,10,11,12,13,14].

Molecular imprinting technology (MIT) can accurately construct spatially and chemically matched recognition sites with target pollutants through template molecular pre-assembly strategies [5] and has demonstrated unique advantages in the fields of sensing and separation, as shown in Figure 1 [6,7,8,9,10,11,12,13,14]. However, traditional molecularly imprinted polymers (MIPs) suffer from defects such as poor photostability and low mass-transfer efficiency, which limit their application in photocatalysis. For this reason, researchers have combined MIT with nanosemiconductor materials, among which TiO_2_ is an ideal carrier due to its high chemical stability, excellent photocatalytic activity, and low cost [15]. The molecularly imprinted TiO_2_ (MI-TiO_2_) prepared by surface imprinting, sol-gel modification, and other strategies not only retained the efficient separation of photogenerated carriers of TiO_2_ but also could target enrichment of the target antibiotics in complex matrices by specifically recognizing the cavities, thus breaking the bottleneck of the efficiency of the traditional photocatalysis for low concentrations of pollutants [16].

This bifunctional synergistic mechanism of “molecular recognition-photocatalytic oxidation” has demonstrated unique advantages in marine pollution management; for example, TiO_2_-functionalized biochar composites developed by Louros et al. were able to significantly improve the efficiency of photocatalytic degradation of sulfadiazine in mariculture wastewater [17]; Qin et al. designed Fe_3_O_4_@TiO_2_-type magnetic molecularly imprinted nanoparticles for efficient and selective adsorption of chloramphenicol in high-salt marine sediments, with recoveries of 77.9–102.5% [18]. The above case validates the engineering potential of MI-TiO_2_ in complex marine environments. In this paper, the synthesis strategy of MI-TiO_2_ and its performance optimization mechanism in marine antibiotic degradation are systematically reviewed, focusing on the influence of modification means such as heterogeneous structure building, elemental doping, and photo-thermal synergism on the photocatalytic performance, and summarizing the removal effect of MI-TiO_2_ on various types of antibiotics with the aim of providing theoretical support for the engineering application of MI-TiO_2_.

## 2. Sources and Characterization of Antibiotics in the Marine Environment

### 2.1. Sources of Marine Antibiotics

Land-based inputs are the main sources of antibiotics in the marine environment, mainly including aquaculture, agricultural activities, the pharmaceutical industry, medical wastewater, and domestic sewage. In aquaculture, antibiotics are widely used for the prevention and treatment of diseases in aquatic organisms and enter rivers or coastal waters through aquaculture wastewater discharged into sewage treatment sites [19]. In agricultural activities, antibiotics enter rivers through agricultural runoff and soil infiltration and eventually enter into the ocean [20]. Pharmaceutical industry wastewater contains high concentrations of antibiotic residues, even up to mg∙L^−1^ of antibiotics in the wastewater discharged by certain companies, which enter the ocean through rivers after treatment [21]. Antibiotics in medical wastewater enter the sewage system through patient excretion, and some residual drugs enter the water body after treatment, and medical wastewater contains more than 25% antibiotics compared to other wastewater [22]. Antibiotic metabolites from domestic wastewater may also enter the marine environment through the sewage system, and this type of wastewater contains the largest number and variety of microorganisms, which increases the emergence of drug-resistant organisms [23]. Existing antibiotic treatment processes have been reported to remove only 36–79% of antibiotics, and residual antibiotics enter the surface water environment through wastewater treatment plant outfalls and ultimately into bays and nearshore environments, which puts tremendous pressure on the marine ecosystem [24].

Atmospheric deposition is another important pathway for antibiotics to enter the oceans and includes both atmospheric wet deposition and atmospheric dry deposition. Atmospheric wet deposition refers to the entry of antibiotics into the atmosphere through aerosols or particulate matter, and then into the marine environment along with rainfall (e.g., rain, snow). Atmospheric dry deposition, on the other hand, involves antibiotics attaching to atmospheric particulate matter and settling directly on the ocean surface by gravity [25]. Both of these methods allow antibiotics to be transported from long distances and enter the ocean, further expanding the scope of their contamination.

Marine inputs mainly include mariculture wastewater and near-shore sewage discharges. In mariculture, antibiotics are used directly in aquaculture waters, and some drug residues are discharged into the ocean through wastewater. Industrial wastewater, domestic sewage, and ship discharges from near-shore areas may also contain antibiotic residues that enter the marine environment directly. These sources make antibiotics widely distributed in the near-shore and pelagic environments, posing a potential threat to marine ecosystems [26].

**Figure 2:**
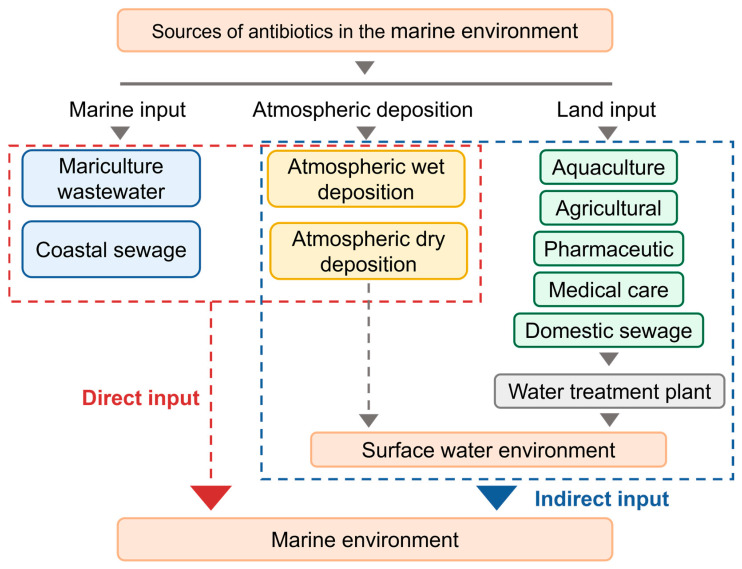
Sources of antibiotics in the marine environment.

Antibiotic contamination in the marine environment is a complex multi-source problem (Figure 2), and together these sources contribute to the widespread distribution of antibiotics in the marine environment, posing a serious threat to marine ecosystems and human health [27]. The levels of antibiotics in marine environments around the world are shown in Table 1. Antibiotics are prevalent in marine environments, with sulfonamides and macrolides being the most commonly detected antibiotics (concentrations ranging from 0.3 to 16,000 ng∙L^−1^), and there are differences in the types of antibiotics and the concentration levels of antibiotics detected in different places, which may be related to the local socioeconomic development level [28]. Therefore, strict control of antibiotic sources and the development of efficient treatment technologies are key to solving the problem of marine antibiotic pollution.

### 2.2. Characteristics of Marine Antibiotics

Antibiotics in the marine environment are a class of organic compounds with complex chemical structures. They usually contain functional groups such as hydroxyl, amino, and carboxyl groups. These groups not only give them biological activity but also significantly affect their migration and transformation behavior in the aquatic environment. The physicochemical properties of different types of antibiotics are significantly different; for example, tetracycline antibiotics are prone to photolysis due to their high water solubility and photosensitivity, while sulfonamide antibiotics can remain in the environment for a long time due to their strong chemical stability [41]. In addition, antibiotics in the environment make it easy to generate more toxic or more difficult to degrade intermediates (e.g., nitro or halogenated derivatives) through hydrolysis, oxidation, and other reactions, and their persistence and bioaccumulation pose multiple threats to marine ecosystems and human health. It is worth noting that the particularity of the marine environment further aggravates the difficulty of antibiotic removal; high salinity (e.g., Cl^−^, SO_4_^2−^) may inhibit the exposure of catalytic active sites through an ion shielding effect or compete with antibiotics for adsorption; coexisting pollutants (e.g., heavy metals, microplastics, and petroleum hydrocarbons) not only interfere with the targeted identification of antibiotics but also may amplify ecological risks through synergistic toxic effects [42].

### 2.3. Traditional Methods of Antibiotic Removal

Conventional treatment methods for antibiotic pollution in the marine environment mainly include physical, chemical, and biological technologies, but each has significant limitations. Although physical methods such as adsorption (activated carbon, clay, etc.) and membrane filtration (nanofiltration, reverse osmosis) can effectively retain antibiotics, they only achieve pollutant transfer rather than degradation and face difficulties in adsorbent regeneration, membrane fouling, and high energy consumption [43]. In chemical methods, although advanced oxidation processes (such as Fenton oxidation and ozone oxidation) can degrade antibiotics through reactive oxygen species (ROS), they often require harsh reaction conditions (e.g., specific pH) and may produce toxic by-products (e.g., halogenated compounds) [44]. Chlorination disinfection is widely used, but it is easy to produce more toxic chlorinated derivatives. Biodegradation depends on the activity of specific microorganisms or enzymes, but its efficiency is limited in high-salt and low-temperature marine environments and may accelerate the spread of antibiotic resistance genes (ARGs) [45]. In contrast, photocatalytic technology (especially TiO_2_-based materials) shows significant advantages; it mineralizes antibiotics into CO_2_, H_2_O, and harmless intermediates through photogenerated ROS (e.g., ∙OH, ∙O_2_^−^), which is both efficient and environmentally friendly [46]. The characteristics of solar energy drive reduced energy consumption, in line with the principle of green chemistry; through functional modification such as element doping and heterostructure construction, it can adapt to high-salt or turbid seawater environments [47]. Moreover, MI-TiO_2_ can achieve efficient degradation of antibiotics under low-concentration conditions, providing a new generation of solutions for marine antibiotic pollution control.

## 3. Preparation and Modification of Imprinted Titanium Dioxide Catalysts

### 3.1. Preparation Method

#### 3.1.1. Surface Molecular Imprinting

**Figure 3:**
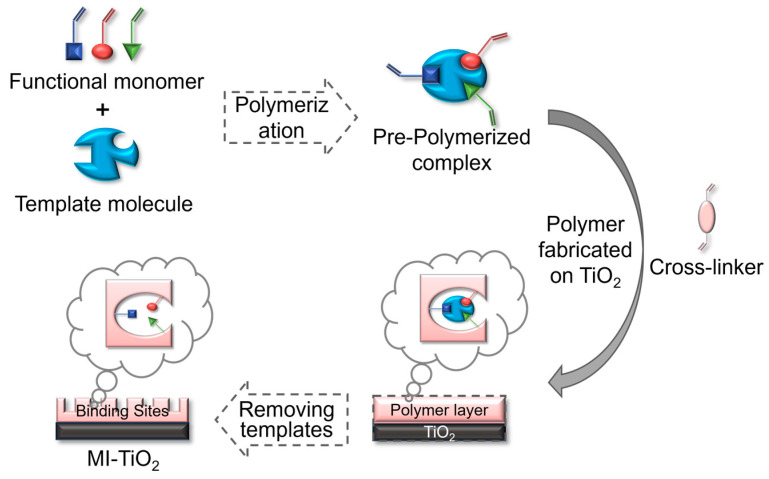
Synthesis process of surface MI-TiO_2_ [48].

Surface molecular imprinting is a more commonly used method that refers to an imprinting technique in which a polymerization reaction occurs on the surface of a carrier, such as a chemically modified silica gel or metal oxide, so that the molecularly imprinted recognition sites are distributed on the surface of the molecularly imprinted polymer or solid-phase matrix. Combining the ability of TiO_2_ photocatalytic degradation of organic pollutants and the ability of molecularly imprinted polymers to recognize organic pollutants exclusively, by selecting suitable functional monomers and template molecules, and then adding cross-linking agents and titanium sources as precursors, specific pores were formed on the surface of TiO_2_ through chemical polymerization reactions to generate pre-polymerized complexes, which can achieve selective degradation of high-toxicity and low-concentration pollutants (Figure 3) [48]. The molecular imprinting on the TiO_2_ surface, on the one hand, can expose the molecular recognition sites on the TiO_2_ surface and improve the mass transfer between the recognition sites and the target molecules; on the other hand, the template molecules are easy to be eluted completely, and it is favorable for the recombination between the recognition sites and the template molecules. In addition, the catalytic performance of the composites can be improved by adjusting the synthesis parameters, such as reaction time, temperature, and pH value, so that the composites can perform more efficiently and persistently in the treatment of low concentrations of highly toxic organic pollutants. Li et al. [49] used surface molecularly imprinted technology to prepare TiO_2_/SiO_2_ hybrid fibers by adding templates to the precursor solution, with titanate-butyl ester (TBOT) as the titanium source, and the functional monomer binds to rhodamine B (RhB) and generates specific recognition sites, and the imprinted fibers exhibit higher adsorption capacity and selectivity compared to the non-imprinted samples. He et al. [50] prepared magnetic molecularly imprinted nanocomposites by surface imprinting, which were capable of dispersive solid-phase extraction of doxycycline from marine sediments with a low detection limit (0.03 μg·g^−1^) and excellent recoveries (90.60~93.76%). Li et al. [51] prepared molecularly imprinted Ag_3_PO_4_/TiO_2_ photocatalysts (MATs) using sulfadimethoxine as a template and TBOT as a functional monomer, and the MAT surfaces formed distinct imprinted cavities and n-p-type heterojunctions on the surface of MAT and had a large specific surface area, and the degradation rate of sulfadimethoxine reached 82.4%.

#### 3.1.2. Molecularly Imprinted Sol-Gel Technology

The molecularly imprinted sol-gel technique is a method of introducing template molecules into an inorganic network structure through a sol-gel process, which elutes to form a rigid material. This technique combines the advantages of the sol-gel method, such as mild preparation conditions, modulation of the average pore size, pore distribution, and specific surface area of the material, while overcoming the shortcomings of traditional molecularly imprinted organic polymers that are less rigid and inert [52]. Therefore, molecularly imprinted sol-gel technology has become an important direction of current research, providing a new way for the preparation of high-performance molecularly imprinted materials. Huang et al. [53] synthesized a novel photocatalyst (MIP-Nd-TiO_2_) by the sol-gel method and optimized the preparation conditions by adjusting the doping amount of Nd, calcination conditions, and so on, which resulted in the degradation of oxytetracycline (OTC) by up to 91.97%. Ferreira et al. [54] used titanium isopropoxide (TTIP) and methyltriisopropoxide titanium (MTTIP) as precursors to synthesize methyl-modified hollow TiO_2_ microspheres with selectivity by the sol-gel method and obtained a certain degree of mixed-network organization, which increased with the calcination temperature.

#### 3.1.3. Other Methods

In addition to the above methods, molecularly imprinted TiO_2_ is also prepared by the hydrothermal method and liquid phase deposition method. The hydrothermal method forms molecularly imprinted TiO_2_ materials with specific pore structures by reacting titanium source precursors with template molecules and functional monomers in a high-temperature and -pressure hydrothermal environment using water as the reaction medium [55]. The materials prepared by this method usually have high crystallinity and stability, and the morphology and properties of the materials can be further optimized by adjusting the parameters, such as hydrothermal temperature, time, and pH. Xiong et al. [56] prepared a novel electrochemical sensor (MIPs/TiO_2_ NRAs@FTO) for the detection of salicylic acid by the hydrothermal method. The nanorod structure of TiO_2_ for SA provided high specific surface area, increased imprinting sites, improved internal mass transport, and enhanced the accessibility of the active sites, thus improving the sensitivity and binding ability of the sensor. Liquid phase deposition is a technique to deposit TiO_2_ thin films on the substrate surface by chemical reaction in solution at room temperature or low temperature. This method is simple, low-cost, and enables precise regulation of film thickness and structure. Li et al. [57] used the sol-gel method combined with the liquid phase deposition technique to prepare iron-doped TiO_2_/SiO_2_ (Fe@TS) nanofibrous membranes with molecularly imprinted modification, and the composites formed a thin layer of molecularly imprinted polymers in liquid phase, and 4-nitrophenol’s photodegradation showed excellent selectivity.

### 3.2. Modification Method

Conventional TiO_2_ suffers from a narrow photoresponse range, high photogenerated carrier complexation rate, and poor selectivity to target pollutants, which limit its practical applications. To overcome these limitations, researchers have optimized molecularly imprinted TiO_2_ through various modification methods, including elemental doping, composite structure construction, morphology modulation, surface functionalization, photothermal synergistic effect, and magnetic/electric field-assisted catalysis [58].

#### 3.2.1. Elemental Admixture

Elemental doping is used to enhance photocatalytic efficiency by introducing metallic or non-metallic elements into the TiO_2_ lattice to modulate its electronic structure and light absorption properties. Metal doping (e.g., Fe, Cu, Ag, etc.) introduces impurity energy levels and promotes the separation of photogenerated electron–hole pairs while broadening the photoresponse of TiO_2_ to the visible region. Uthiravel et al. [59] prepared Ag-doped TiO_2_ nanoparticle photocatalysts by the co-precipitation method, and the Ag doping significantly reduced the band gap of TiO_2_ (3.3 eV for TiO_2_, 0.9 eV for Ag-TiO_2_), and the degradation of methylene blue by Ag-TiO_2_ under light was as high as 96.96%. Non-metal doping (e.g., N, C, S, etc.), on the other hand, enhances the light absorption ability of TiO_2_ under visible light by lowering its bandgap. Kuang et al. [60] proposed an N-doped TiO_2_/Ti_3_C_2_ heterojunction-driven auto-photocatalytic platform for the detection of dexamethasone (DXM), where N doping promotes the conversion of dissolved oxygen to H_2_O_2_, providing more co-reactants to enhance the electrical signal and making it so that the molecularly imprinted electrochemical sensor has a wide linear range (1.0 × 10^−6^–1.0 × 10^1^ μg∙mL^−1^) and low detection limit. Elemental doping can effectively improve the photocatalytic activity of TiO_2_ and enhance its ability to degrade the target pollutants, and the effects of different elemental doping on the photocatalytic performance of TiO_2_ are shown in Table 2. These elemental doping strategies provide a new idea for the development of highly efficient and stable MI-TiO_2_ photocatalysts, which is expected to promote its practical application in the treatment of marine antibiotic wastewater.

#### 3.2.2. Composite Structure Construction

Composite structure construction is carried out by combining TiO_2_ with other materials (e.g., semiconductors, carbon materials) to form heterojunctions or composites to enhance its photocatalytic performance. Deng et al. [72] prepared TiO_2_ NP/g-C_3_N_4_ photocatalysts, and the heterojunction formed was able to inhibit the compounding of photogenerated electron–hole pairs effectively to improve the photocatalytic efficiency. Lin et al. [73] prepared composite photocatalysts using P25TiO_2_ and graphene as raw materials to prepare a composite photocatalyst; graphene can broaden the range of photo-response of the material and improve its electrical conductivity, which can help to quickly transfer the electrons generated by the light excitation of TiO_2_ and reduce the electron–hole pair compounding, thus enhancing the photocatalytic activity, and the experimental results showed that a 10 mg∙L^−1^ solution of methyl orange was able to be completely degraded within 12 min, which was 1.81 times that of TiO_2_. Ye et al. [74] prepared a CdS/TiO_2_ composite photocatalyst using a ball milling process, which showed a photocatalytic degradation efficiency of 57.84% for methyl orange after 2 h of UV illumination. Qin et al. [75] prepared TiO_2_/BiYO_3_ photocatalysts for water resolution of hydrogen, and the experimental results showed that the photocatalytic hydrogen precipitation rate was 10 times higher than that of TiO_2_ and 57 times higher than that of BiYO_3_, respectively. Alimard et al. [76] synthesized Bi/BiOBr/TiO_2_ composites by the solvothermal method, which showed high photocatalytic performance for NO and NO_2_ under both visible and UV lamps. The composite structure can achieve efficient photocatalytic degradation by modulating the energy band structure and interfacial properties of the material (Table 3), providing a more efficient solution for environmental treatment and energy conversion.

#### 3.2.3. Conformal Modification

Conformal modulation is used to optimize the photocatalytic performance of TiO_2_ by designing its microstructure (e.g., nanotubes, nanosheets, nanospheres, etc.). Wu et al. [89] prepared TiO_2_ nanotube (TiNT) arrays on titanium foil and synthesized TiO_2_ nanotube arrays with a high specific surface area and ordered structure, which could provide more active sites and enhance the efficiency of photocatalytic reaction. Sharafudheen et al. [90] prepared porous TiO_2_ nanomaterials using titanium isopropoxide as raw material, and the BET analysis results showed a specific surface area of 134 m^2^∙g^−1^, and the excellent specific surface area increased the contact area between the reactants and the catalysts, which further enhanced the degradation effect. Liu et al. [91] prepared Ag/TiO_2_ nanofibrous films, and the synthesized nanofibrous films with a larger specific surface area and more reaction sites were provided, and the degradation rate of rhodamine B reached 73%, which was much higher than that of TiO_2_ particles. The morphology modification not only improved the physicochemical properties of TiO_2_ but also enhanced its adsorption and degradation of target pollutants, providing an efficient catalyst for marine antibiotic wastewater treatment.

#### 3.2.4. Surface Functionalization

Surface functionalization is the process of improving the surface properties of TiO_2_ by introducing organic molecules or functional groups (e.g., carboxylic acids, amine groups, etc.) on its surface to enhance the selective adsorption and degradation of specific pollutants. Mendonça et al. [92] used ethylenediamine-modified activated carbon and impregnated it with TiO_2_ to prepare a novel absorbent/photocatalyst material (AC-ET/TiO_2_), and the insertion of amine groups enhanced the stability of the TiO_2_ surface for rapid degradation of sulfadimethazine. Wu et al. [93] successfully synthesized six functional conjugated microporous polymers (CMPs) containing amino, hydroxyl, carboxyl, and ester groups via the Sonogashira–Hagihara coupling reaction and uniformly encapsulated TiO_2_ on the surface of the CMPs under solvent-heated conditions. Due to the high electronegativity of the carboxyl group, The CMP/TiO_2_ containing carboxyl groups showed the smallest band gap, the highest photocurrent intensity, and the lowest electrical resistance, which significantly improved the photocatalytic activity. Wang et al. [94] prepared an acid-induced assembly of rutile TiO_2_ photocatalysts by treating layered protonated titanates using a concentrated HNO_3_ solution. The experimental results showed that nitrate grafting made the surface of rutile TiO_2_ negatively charged, which was conducive to the capture of positive protons and improved carrier separation, thus enhancing photocatalytic hydrogen production. The surface functionalization not only improves the selectivity of TiO_2_ but also optimizes its interaction with the target pollutants, enabling it to exhibit higher catalytic efficiency in complex wastewater systems.

#### 3.2.5. Photothermal Synergy

The photothermal synergistic effect is achieved by compounding TiO_2_ with photothermal materials (e.g., carbon-based materials, metal sulfides), which utilizes the photothermal effect to enhance the local temperature and accelerate the reaction kinetics. Li et al. [95] successfully prepared black TiO_2_/MoS_2_/Cu_2_S hierarchical tandem heterojunction visible light photocatalysts with a mesoporous structure by evaporation-induced self-assembly, high-temperature hydrogenation, and solvent-thermal method, which can effectively absorb near-infrared energy to enhance the photothermal effect, and the narrow bandgap properties of MoS_2_ and Cu_2_S can efficiently convert sunlight into heat, thus significantly enhancing the photocatalytic performance. Yang et al. [96] prepared photothermally coupled TiO_2_/BiS S-type heterojunction nanofibers for photothermally catalyzed CO_2_ reduction, and the excellent photothermal conversion ability of BiS enabled the heterogeneous photocatalysts to accelerate the photogenerated electron transfer rate, and surface reaction rates were further accelerated, which were 5.24 times higher than those of the pristine TiO_2_. Su et al. [97] designed MOF-derived C/TiO_2_ composites with simultaneous photothermal and photocatalytic functions for wastewater purification, and the materials possessed excellent sunlight absorptivity and superhydrophilicity, a large specific surface area, and a porous structure and degraded bottom organic pollutants in the water by up to 92.75%. The photothermal synergistic effect not only enhances the photocatalytic performance of TiO_2_ but also improves its applicability in complex wastewater systems, which provides a new idea for the treatment of marine antibiotic wastewater.

#### 3.2.6. Magnetic/Electric Field-Assisted Catalysis

Magnetic/electric field-assisted catalysis is the enhancement of photocatalytic performance by compositing TiO_2_ with magnetic materials (e.g., Fe_3_O_4_) using an applied magnetic or electric field. Tang et al. [98] successfully achieved efficient photocatalytic degradation of tetracycline over Ag_2_S/TiO_2_ catalysts using an external magnetic field-assisted strategy due to the fact that the Lorentzian force of an external magnetic field acting on charge carriers can promote the photogenerated carrier separation, which further enhances the catalytic effect. Grzegórska et al. [99] prepared a TiO_2_/Ti_3_C_2_/MnFe_2_O_4_ magnetic photocatalyst, which was able to completely degrade carbamazepine and ibuprofen under simulated sunlight with PMS-assisted photo-degradation in 20 min and could be magnetically separated by an external magnetic field after the degradation process. In Gu et al. [100], Z-type WO_3_/TiO_2_ heterojunction catalysts were successfully prepared by an impregnation sintering process, and 98% degradation was achieved at an initial dichloromethane concentration of 200 ppm, and the excellent performance was mainly attributed to the built-in electric field and narrower bandgap, which effectively reduced the electron and hole complexation and thus increased the generation of more reactive oxygen species. The magnetic/electric field-assisted catalysis not only improves the catalytic performance of TiO_2_ but also enhances its maneuverability and cyclic stability in practical applications, providing an efficient and sustainable solution for marine antibiotic wastewater treatment.

As shown in Table 4, morphology modification, surface functionalization, photothermal synergy, and magnetic/electric field-assisted catalytic modification strategies can significantly improve the catalytic activity of TiO_2_, with breakthroughs in photodegradation efficiency, target selectivity, and energy conversion performance. However, the high salinity of the marine environment, the coexistence of multiple pollutants, and the cost constraints of engineering applications require the optimization of the modification schemes for specific scenarios in order to promote large-scale applications. It should be noted that each modification strategy has significant limitations while enhancing the performance (Table 5), which should be balanced and optimized according to actual needs.

## 4. Application of Imprinted Titanium Dioxide in Antibiotic Degradation

**Figure 4:**
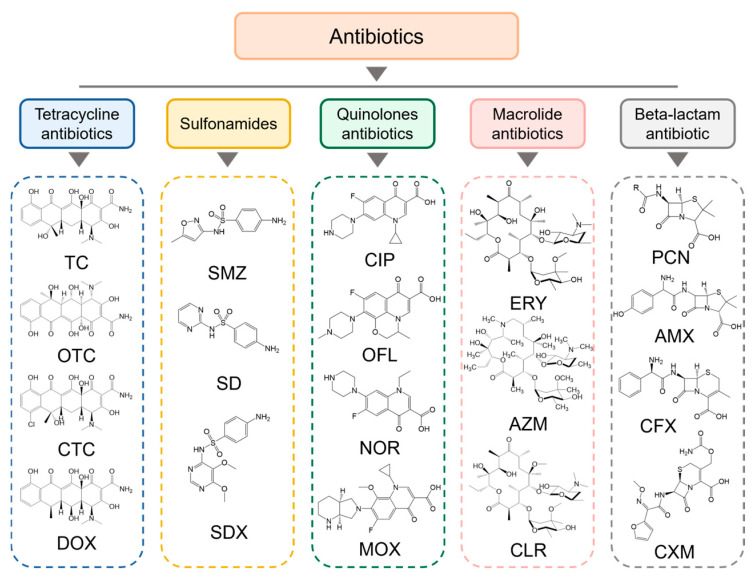
Antibiotics commonly found in the marine environment.

**Figure 5:**
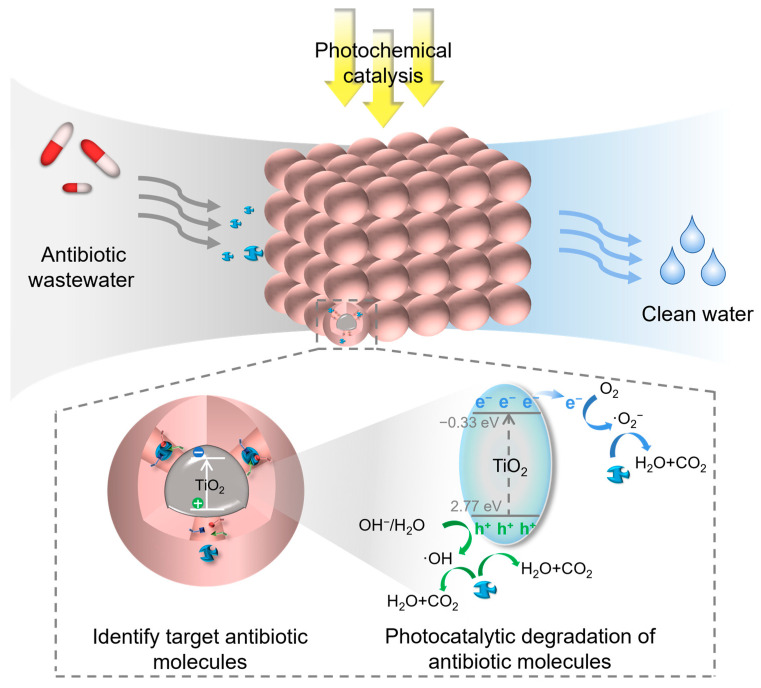
Mechanism of antibiotic degradation by MI-TiO_2_.

In recent years, antibiotic pollution has become a hot issue studied by scholars from various countries, especially the fact that only 15% of antibiotic drugs will be absorbed and utilized, and the rest will be directly discharged into the ecosystem in the form of prodrugs, which has a very serious impact on the environment [108]. In addition, antibiotics are characterized by many interfering factors and low residues, so it is very important to choose a suitable treatment method [109]. MI-TiO_2_ photocatalytic degradation of antibiotics is an efficient technology for environmental treatment. Its mechanism is based on the catalyst design of molecular imprinting technology, which can selectively recognize and adsorb specific antibiotic molecules to achieve antibiotic degradation efficiency under low-concentration conditions [110]. Common antibiotics, as shown in Figure 4, are mainly classified into tetracyclines (e.g., tetracycline, oxytetracycline), sulfonamides (e.g., sulfamethoxazole, sulfadiazine), quinolones (e.g., ciprofloxacin, norfloxacin), macrolides (e.g., erythromycin, azithromycin), and *β*-lactams (e.g., penicillin, cephalosporin). The degradation mechanism can be summarized as follows (Figure 5): (1) molecularly imprinted cavities target the adsorption of target antibiotics and enrich the pollutants through specific interactions (e.g., hydrogen bonding, electrostatic, or hydrophobic interactions); (2) photoexcitation of TiO_2_ generates electron–hole pairs, generating reactive oxygen species (ROS) such as hydroxyl radicals (∙OH) and superoxide radicals (∙O_2_^−^), which leads directly to oxidative degradation of antibiotic molecules [111,112]. The imprinted cavity can effectively inhibit the photogenerated carrier complexation and prolong the contact time between the active species and the pollutants, thus improving the catalytic efficiency, which is divided into the following studies for the degradation of five types of typical antibiotics.

### 4.1. Tetracycline Antibiotics

Tetracycline antibiotics (TCs) are difficult to be effectively degraded by conventional processes due to the chemical inertness of their benzotetracycline rigid skeleton. MI-TiO_2_ significantly enhanced the selective capture of trace tetracycline antibiotics by precisely designing the imprinted cavities matching the hydroxyl and amide groups of the tetracycline antibiotics. Li et al. [113] combined the Stöber method with the sol-gel method and successfully constructed the molecularly imprinted molecules with core–shell structure TiO_2_ (MIP-TiO_2_@SiO_2_), whose imprinting sites were highly compatible with the molecular conformation of tetracycline, and the degradation rate of tetracycline reached 82.18% under 60 min visible light irradiation. Huang et al. [53] optimized the energy bands of the MIP-Nd-TiO_2_ catalyst energy band structure; the degradation rate of oxytetracycline was as high as 91.97% under simulated sunlight, and the degradation pathway was revealed by LC-MS analysis: *β*-keto group demethylation → aromatic ring breakage → final mineralization to CO_2_ and H_2_O. The surface-imprinted photocatalyst (TMIP) developed by Fu et al. [114] effectively overcame the constraints of reaction kinetics. Under visible light irradiation, complete tetracycline degradation was achieved within 20 min (k = 0.153 min^−1^), with a selectivity coefficient for tetracycline antibiotics (α = 3.367) significantly exceeding those for ciprofloxacin (2.389) and sulfonamide pollutants (<1.2). These results confirm that the conformationally-matching imprinted cavities selectively enhance tetracycline degradation through structural recognition. Fu et al. [115] prepared an efficient composite photocatalyst by adjusting the doping ratios of different metal ions, and the degradation efficiency of tunicamycin in mariculture wastewater was as high as 73.04%. Guo et al. [116] used a Si-doped molecularly imprinted material prepared by liquid phase deposition method (TiO_2_/SiO_2_/OTC), which showed 80.79% degradation of oxytetracycline under 120 min xenon lamp irradiation through the domain-limiting effect of the SiO_2_ mesoporous structure and the synergistic effect of charge transfer by the Si-O-Ti bonds, and the activity was stabilized after several cycles. These studies provide theoretical and technological paradigms for the targeted identification and efficient mineralization of tetracycline antibiotics in complex water bodies.

### 4.2. Sulfonamide Antibiotics

Sulfonamide antibiotics (SAs) possess broad-spectrum antimicrobial properties due to their sulfonamide moiety and p-aminobenzene ring structure, but the chemical inertness of their aromatic rings and the high stability of their sulfonamide bonds lead to persistent residues in the environment, which aggravate the ecological risks. In order to break through this bottleneck, researchers have developed highly efficient targeted degradation systems by molecularly imprinted technology. Li et al. [117] systematically prepared four molecularly imprinted photocatalysts (MIP-TiO_2_/SD, MIP-TiO_2_/SMZ, MIP-TiO_2_/SN, and MIP-TiO_2_/AN) and found that the selective degradation efficiencies of sulfadiazine and sulfamethoxazole differed significantly from those of sulfonamide and sulfamethoxazole. The selective degradation efficiencies differed significantly, and the following common degradation pathways of sulfonamide antibiotics were revealed by intermediates analysis: single bond breaking, hydroxyl radical (∙OH) mediated ring-opening reaction, and oxidative removal of amino groups. Wang et al. [118] constructed an Fe-doped molecularly imprinted photocatalyst (SA-Fe@TiO_2_) in one step, with photogenerated holes (h^+^), ∙OH, and superoxide radicals (∙O_2_^−^) synergistically enhancing the sulfamethoxazole degradation rate by 4.3-fold compared with pure TiO_2_, while exhibiting excellent ability to inactivate antibiotic resistance genes, significantly reducing the risk of environmental drug resistance spread. Zhang et al. [119] designed a novel ternary composite catalyst (MFTC, TiO_2_@Fe_2_O_3_@g-C_3_N_4_) whose molecularly imprinted cavity’s ability to target captured sulfamethoxazole resulted in a degradation rate up to twice that of similar pollutants and stable performance after several cycles. These studies deepened the mechanism of selective photocatalytic degradation of sulfonamide antibiotics from molecular recognition/active site modulation/interfacial charge separation in a multi-dimensional way, which provides a new idea for the precise management of sulfonamide pollutants in complex water bodies.

### 4.3. Quinolone Antibiotics

The fluoroquinolone structures of quinolone antibiotics (QNs) have strong antimicrobial activity, but their chemical stability increases the difficulty of environmental degradation. Researchers have developed several efficient degradation strategies by molecular imprinting technology (MIT). Li et al. [120] prepared TiO_2_-loaded carbon nanosheet composites using ciprofloxacin as a template molecule. The material significantly enhanced the targeted degradation efficiency of quinolone antibiotics at low concentrations through the synergistic effects of the specific recognition of molecularly imprinted cavities, the adsorption enhancement of the carbon matrix material, and the photocatalytic activity of TiO_2_, with an adsorption selectivity coefficient of 7.2 and a photocatalytic selectivity coefficient of 3.2 for ciprofloxacin. Qin et al. [121] used molecularly imprinted polymers for the selective detection of norfloxacin in seawater, with detection limits of 2 µg·L^−1^ and 5 µg·kg^−1^ in seawater samples and sediments, respectively. Zheng et al. [122] further designed a mesoporous TiO_2_-based inorganic molecularly imprinted magnetic photocatalyst (MIFTA) whose unique pore structure and magnetic components endowed the material with high selectivity (norfloxacin adsorption capacity of 135.7 µg∙mg^−1^, which was 1.4–2.3 times higher than that of conventional catalysts) and convenient recyclability (magnetic separation efficiency > 95%). In addition, Li et al. [123] modified commercial TiO_2_ particles (P25) with a surface molecular imprinting technique, and the resulting imprinted material showed significantly better adsorption capacity for norfloxacin than non-imprinted material and pristine P25, and the removal efficiencies for structurally similar ciprofloxacin, carbamazepine, and phenol were 78.87%, 7.87%, and 2.68%, respectively, which sufficiently verified that molecularly imprinted sites are effective for fluoro quinolones by the specific affinity mechanism of molecularly imprinted sites. These studies provide important theoretical and technical support for the efficient and selective removal of quinolone antibiotics in complex water bodies.

### 4.4. Macrolide Antibiotics

Macrolide antibiotics (MLs) can be efficiently bound to the specific cavities of MI-TiO_2_ through intermolecular forces such as hydrophobic interaction, hydrogen bonding, and π-π stacking. To address its environmental residue problem, Xie et al. [124] developed an erythromycin molecularly imprinted polymer (EMIP), which has a mesoporous structure (specific surface area of 265.62 m^2^∙g^−1^, pore size of 2–5 nm) and hydrophobic surface properties, with an adsorption capacity of 4015.51 μg∙g^−1^ at an erythromycin concentration of 100 μg∙L^−1^ and adsorption efficiency remaining above 80% after five cycles. The retention of performance was >80%, attributed to the precise matching of the imprinted cavity to the erythromycin macrolide skeleton. In the field of photocatalytic degradation, Čizmić et al. [125] demonstrated a broad-spectrum applicability of TiO_2_ nanofilms prepared by the over sol-gel method, evaluated the effects of different pH, aqueous substrate, drug coexistence, and radiation source factors on the degradation process and identified five azithromycin degradation products, none of which showed toxicity, suggesting the effective removal of azithromycin. Satulu et al. [126] counted a CA-GO-TiO_2_/PTFE composite membrane with a gradient pore structure, in which the polytetrafluoroethylene (PTFE) substrate successfully maintained the bulk porosity of the carrier while improving the thermal and chemical stability of the membrane, and the degradation of azithromycin effluent was more than 80%, which provided a technical support for the large-scale treatment of macrolide antibiotics.

### 4.5. β-Lactam Antibiotics

*β*-lactam antibiotics (BLs) exert potent antimicrobial effects through their characteristic *β*-lactam ring; however, the high reactivity of this four-membered ring leads to its susceptibility to non-selective hydrolysis in environmental media, generating eco-toxic ring-opening derivatives (e.g., phenylacetic acid metabolites). To address the above problems, researchers have developed a variety of targeted processing techniques. Wang et al. [127] designed a carbon quantum dot-functionalized molecularly imprinted polymer (CPDs-NH@MIP) whose surface-modified amino group and conjugated double bond can accurately recognize ceftiofur sodium molecules through multiple interactions (hydrogen bonding, π-π stacking) and break through the spatial site resistance limitation of traditional adsorbents; an equilibrium adsorption of 68.62 mg∙g^−1^ with a selectivity coefficient of 5.61 was achieved in 10 min. In the field of catalytic degradation, Mehralipour’s team [128] constructed rGO/Fe^0^/Fe_3_O_4_/TiO_2_ nanocomposites with hierarchical porous structure by the sol-gel method, which showed a degradation rate of 96% of the penicillin solution of 52 mg∙L^−1^, and the catalyst can be recovered by an applied magnetic field. Further, the TiO_2_/Bi_2_MoO_6_ heterojunction photocatalyst constructed by Wang et al. [129] exhibited significantly enhanced photocatalytic performance during the degradation of amoxicillin, with reaction rate constants that were 18.2 and 5.7 times higher than those of pure TiO_2_ and Bi_2_MoO_6_, respectively, which were attributed to the Z-type heterojunction-promoted directional mobility of the photogenerated carriers and the enhanced interfacial charge separation efficiency.

Table 6 summarizes the performance of MI-TiO_2_ on five major antibiotics in marine wastewater. The excellent selectivity coefficients and degradation rates confirm that the precise matching between the imprinted cavities and the molecular structures of pollutants can effectively avoid the interference of the coexisting pollutants’ competitive adsorption, which highlights the potential of the engineering application of MI-TiO_2_ in complex marine environments.

## 5. Conclusions and Outlook

Molecularly imprinted TiO_2_ (MI-TiO_2_) exhibits high efficiency (>90%) and selectivity in the targeted degradation of marine antibiotic pollutants (such as sulfonamides and tetracyclines) by combining molecular imprinting technology (MIT) with the photocatalytic properties of TiO_2_, and the convenient recovery of the catalyst is realized by magnetic field-assisted technology. However, its practical application still faces multiple challenges: the uniformity of imprinting sites and the efficiency of template elution need to be optimized in the industrial amplification process, and the mechanism of material stability and activity attenuation in long-term recycling needs to be analyzed. In addition, photocatalytic degradation may generate toxic intermediates (such as chlorinated or nitrified derivatives). It is necessary to clarify its environmental risks through LC-MS analysis and ecotoxicity assessment and to develop enhanced oxidation or biological synergistic processes to achieve complete mineralization. In terms of industrial scalability, the current synthesis cost is high (involving template molecule preparation, dopant introduction, etc.), and it is necessary to explore low-cost biomass templates and green synthesis routes to reduce large-scale production costs. The following recommendations can be made for future investigations:

- A photo-electro-magnetic synergistic catalytic system was constructed to improve the degradation efficiency and monitor the formation of by-products in real time to ensure environmental safety.
- Artificial intelligence is used to optimize the geometric configuration and doping strategy of imprinted cavities, so as to realize the efficient recognition and degradation of specific antibiotics.
- The long-term performance of MI-TiO_2_ in complex environments was evaluated by establishing a test platform that simulated real marine conditions (such as dynamic salinity and biofouling).
- The full-cycle environmental footprint of MI-TiO_2_ from synthesis to abandonment was systematically analyzed to promote the development of sustainable technology.

Through interdisciplinary technological innovation and large-scale engineering practice, MI-TiO_2_ is expected to become an efficient, low-toxic, and recyclable core solution for marine pollution control, helping to achieve the synergistic goal of ecological restoration and sustainable development.

## Figures and Tables

**Table 1:** Levels of antibiotics in the marine environment worldwide.

Sea (Country)	Antibiotic	Concentration (ng∙L^−1^)	Reference
Beibu Gulf (China)	Sulfamethoxazole	0–15.9	[29]
	Trimethoprim	0–4.11	
	Erythromycin	2.59–47.6	
Bohai Bay (China)	Tetracyclines	41.5–222.4	[30]
Maowei Sea (China)	Demeclocycline	276 ± 71.6	[31]
	Norfloxacin	1.56 ± 1.46	
	Enrofloxacin	0.85 ± 0.65	
Hailing Island (south coast of England)	Oxytetracycline	0–16,000	[32]
	Trimethoprim	0–20	
North coast of the Persian Gulf (Iran)	Norfloxacin	1.21–51.5	[33]
Baltic Sea (Northern Europe)	Sulfamethoxazole	0–311	[34]
	Trimethoprim	0–279	
Po Valley (Italy)	Clarithromycin	0–128.1	[35]
	Ciprofloxacin	0–124	
Cadiz Bay (Spain)	Azithromycin	0–1.2	[36]
	Erythromycin	0–0.3	
South Sea (Korea)	Norfloxacin	0–0.5	[37]
	Lincomycin	0–438	
Red Sea (Saudi Arabia)	Sulfamethoxazole	31.5–62.4	[38]
	Metronidazole	51.0–178.6	
Eastern Mediterranean (Greece)	Clarithromycin	0–1.5	[39]
	Amoxicillin	0–127.8	
Chesapeake Bay (United States)	Azithromycin	0–2.7	[40]
	Norfloxacin	0–94.1	

**Table 2:** Effect of doping with different elements on the photocatalytic performance of TiO_2_.

Element	Photocatalyst	Pollutant	Degradation (%)	Reference
Pr	Pr-MIP-TMCs	Dinitrophenol	92	[61]
Ni and F	Ni-F-TiO_2_	acetaminophen	84	[62]
P	0.071PT	*Escherichia coli*	90	[63]
Ag and Zn	Ag/Zn-MIP-TiO_2_	Ethyl hydroxybenzoate	99	[64]
Ce	Ce-TiO_2_	Tetracycline	86	[65]
K	TNT-K5	Methylene blue	97	[66]
La	La/TiO_2_	Cyanide	98	[67]
B	B-TiO_2_	Diclofenac sodium	98	[68]
Mg	Mg-doped TiO_2_	Methyl orange	95	[69]
V	(TiO_2_:V)/rGO	Rhodamine B	95	[70]
La and I	LICT	Methylene blue	98	[71]

**Table 3:** Effect of different composites on the photocatalytic performance of TiO_2_.

Material	Photocatalyst	Pollutant	Degradation (%)	Reference
CQDs	TiO_2_/CQDs	Methyl orange	85	[77]
LaFeO_3_	LaFeO_3_/TiO_2_	Methylene blue	96	[78]
Chitosan	TiO_2_/Chitosan	Gallic acid	81	[79]
MoS_2_	TiO_2_/MoS_2_	Oilfield suspended solids	93	[80]
FeOOH	FeOOH/TiO_2_	Rodamine B	84	[81]
Bi_2_O_3_	Bi_2_O_3_/brookite TiO_2_	Ofloxacin	91	[82]
BiPO_4_	TiO_2_/BiPO_4_	Kamasipin	88	[83]
Activated Charcoal	AC-TiO_2_	N-Acetyl-p-Aminophenol (APAP)	82	[84]
g-C_3_N_4_	g-C_3_N_4_-TiO_2_-Ag	Malachite green	66	[85]
ZnO and rGO	ZnO-TiO_2_/rGO	Methylene blue	100	[86]
MoS_2_	BC/MoS_2_/TiO_2_	Escherichia coli	100	[87]
Ag_2_CrO_4_	Ag_2_CrO_4_/TiO_2_	NO_2_^−^	100	[88]

**Table 4:** Effects of different modification methods on the photocatalytic performance of TiO_2_.

Method	Photocatalyst	Target Substance	Photocatalytic Performance	Reference
Conformal modification	CR- TiO_2_ NPs	Phenol red	Degradation rate 94%	[90]
	Ag/TiO_2_ nanofiber film	Rhodamine B	Degradation rate 73%	[91]
Surface functionalization	AC-ET/90TiO_2_	Sulfadimethoxine	Degradation rate 90%	[92]
	CMP/TiO_2_	Ciprofloxacin	Degradation rate 97%	[93]
	Rutile TiO_2_	Hydrogen production	Hydrogen precipitation rate 402 μmol·h^−1^	[94]
Photothermal Synergy	TiO_2_/MoS_2_/Cu_2_S	Hydrogen production	Hydrogen precipitation rate 3377 μmol·h^−1^	[95]
	TiO_2_/BiS	CO_2_ reduction	Reduction rate 8 μmol·h^−1^	[96]
	UiO-66-NH_2_(Ti)	Methyl orange	Degradation rate 93%	[97]
Magnetic/Electric field assisted catalysis	Ag_2_S/TiO_2_	Tetracycline	Degradation rate 96%	[98]
	TiO_2_/Ti_3_C_2_/MnFe_2_O_4_	Ibuprofen	Degradation rate 100%	[99]
	WO_3_/TiO_2_	Dichloromethane	Degradation rate 98%	[100]

**Table 5:** Advantages and disadvantages of different modification methods.

Modification Method	Advantages	Disadvantages	Reference
Element doping	High carrier separation efficiency; high selectivity	Doping amount is difficult to control; high cost	[101]
Composite structure construction	Versatility; high stability	Complicated preparation process; difficult to recover	[102]
Conformal modification	High surface area; strong adsorption properties	Difficult preparation; poor structural stability	[103]
Surface functionalization	High selectivity; high dispersibility	Poor modification stability; side reactions	[104]
Photothermal synergistic effect	High reaction rate; strong light absorption	High energy consumption; high material cost	[105]
Magnetic/electric field assisted catalysis	High separation and recovery; high reaction efficiency	High energy consumption; limited scope of application	[106,107]

**Table 6:** Comparison of MI-TiO_2_ performance indices for five types of antibiotics.

Antibiotic Type	Antibiotic	Photocatalyst	Selectivity Factor	Degradation (%)	Reference
Tetracycline antibiotics	Tetracycline	MIP-TiO_2_@SiO_2_	-	82	[113]
	Oxytetracycline	MIP-Nd-TiO_2_	1.7	92	[53]
	Tetracycline	TMIP	3.4	100	[114]
	Oxytetracycline	TiO_2_/SiO_2_/OTC	-	81	[116]
Sulfonamide antibiotics	Sulfamethoxazole	MIP-TiO_2_/SMZ	4.0	99	[117]
	Sulfadiazine	MIP-TiO_2_/SD	1.3	95	[117]
	Sulfamethoxazole	MFTC	2.8	97	[119]
Quinolone antibiotics	Ciprofloxacin	CT-MI	3.2	86	[120]
	Norfloxacin	MIFTA	3.1	88	[122]
	Norfloxacin	MIPs	3.4	77	[123]
Macrolide antibiotics	Erythromycin	EMIP	2.6	80	[124]
	Azithromycin	CA-GO-TiO_2_/PTFE	-	80	[126]
*β*-lactam antibiotics	Ceftiofur sodium	CPDs-NH@MIP	5.6	82	[127]
	Penicillin	rGO/Fe^0^/Fe_3_O_4_/TiO_2_	-	96	[128]
	Amoxicillin	TNBM-80	-	95	[129]

## Data Availability

No new data were created or analyzed in this study.
